# Kawasaki Disease Complicated With Macrophage Activation Syndrome: Case Reports and Literature Review

**DOI:** 10.3389/fped.2019.00423

**Published:** 2019-11-01

**Authors:** Ping Jin, Yong Luo, Xi Liu, Jinji Xu, Chunyi Liu

**Affiliations:** ^1^Pediatric Intensive Care Unit, Baoan Maternal and Child Health Hospital, College of Medicine, Jinan University, Shenzhen, China; ^2^Department of Cardiology, Baoan Maternal and Child Health Hospital, College of Medicine, Jinan University, Shenzhen, China

**Keywords:** Kawasaki disease, complication, macrophage activation syndrome, coronary artery, children

## Abstract

Macrophage activation syndrome (MAS) is a rare and severe complication of Kawasaki disease (KD). The clinical feature, early diagnosis and treatment options, and prognosis need to be further determined in patients with KD complicated with MAS. In this report, we retrospectively analyzed three KD patients complicated with MAS who were treated in pediatric intensive care units (PICU) and reviewed the relevant literatures. We find that being male, being age over 2 years old, incomplete KD, intravenous immunoglobulin (IVIG) non-responder, or persistent fever greater than 10 days are all highly associated with occurrence of MAS. Additional work-ups should be performed promptly in patient with above predisposing factors to rule out complication of MAS. Patients with KD complicated with MAS are at a higher risk of having coronary artery involvement or aneurysm formation, which can be reversed with timely treatment. Early identification and prompt treatment are key points for improving the prognosis of KD patients complicated with MAS.

## Introduction

Kawasaki disease (KD), also known as mucocutaneous lymph node syndrome, is one of the most common forms of vasculitis in children (1). When untreated, manifestations of acute inflammation and fever can last on average of 12 days (2), and may also manifest in a variety of complications including coronary artery aneurysm which has a reported incidence rate of up to 25% in untreated KD patients (3, 4). However, the incidence of aneurysm formation decreases to 3–5% in KD cases following IVIG treatment. The other non-cardiac complications include shock, renal diseases, gastrointestinal involvement, macrophage activation syndrome (MAS), sensory nerve deafness (5–8). MAS is a rare and severe complication of KD with an incidence of 1.1–1.9% of all KD patients (9, 10).

MAS is a type of hemophagocytic lymphohistiocytosis (HLH) that occurs under the circumstance of rheumatic diseases and is a life-threatening excessive immune activation syndrome. It is commonly seen in systemic-onset juvenile idiopathic arthritis (sJIA), with incidence rates as high as 8–17% and is also reported in pediatric patients with KD. There were only 8 cases of KD patients complicated with MAS reported between 1979 and 2007 on MEDLINE (11). As of September 2016, the case report of KD complicated with MAS have increased to 69 cases, but only 34 of them had data to confirm the diagnosis of MAS (12). Therefore, MAS is still a rare entity compared to other complications in patients with KD, with an occurrence rate less than 2% (7). Of interesting note, the incidence rate of coronary artery abnormalities in KD complicated with MAS is as high as 46%, which is much higher than that of KD patients which resolve on its own (15–25%) (12).

This report retrospectively analyzes three KD patients complicated with MAS who were treated in the pediatric intensive care unit (PICU) of our hospital and reviews the relevant literature in order to further our understanding of this disease and make an earlier diagnosis of this complication to improve prognosis through timely treatment.

## Case Report

### Case 1

A 4 year and 8-month-old girl initially presents to us with 6 days of fever with Tmax 40°C and 2 days of dysmorphic skin rash. She has also been having chills, intermittent abdominal discomfort, and occasional cough during this period of time. Physical exam notable for cervical lymphadenopathy (between 1.5 and 2.0 cm), strawberry tongue and fissure lips, diffuse maculopapular rash. The diagnosis of incomplete KD was suspected. On the third day of admission, a cardiac ultrasound showed coronary artery dilatation with the left main coronary artery (LMCA) 3.0 mm (Z-score +2.50), and the right coronary artery (RCA) 2.69 mm (Z-score +2.39) (Z-scores were calculated via http://www.tspc.org.tw:80/service/z_score.asp) in diameter. The influenza virus FLUL-A antigen was positive (+). The patient was treated with aspirin (30 mg/kg/day) (13) and a single dose of intravenous immunoglobulin (IVIG) at 2 g/kg infusion within 12 h after above finding on cardiac ultrasound. Fever persisted 36 h after completing IVIG treatment. The patient was deemed IVIG-nonresponsive after a second dose of IVIG (2 g/kg) was administrated and fever, skin rash, and hepatosplenomegaly continued to be noted with little or no improvement. On the sixth day of admission, laboratory data showed platelets at 85 × 10^9^/L, ferritin at 10,615 ng/ml, and triglycerides at 3.42 mmol/L. Hemophagocytic phenomenon was not detected by cytology from bone marrow aspiration, but hemophagocytosis is indicated in her cervical lymph node biopsy. Therefore the patient was diagnosed KD complicated with MAS (HLH-2004) and was initiated on methylprednisolone pulse treatment at 30 mg/kg.d for 3 days, followed with chemotherapy (VP-16) according to the HLH-2004 protocol, with a total treatment course of 11 weeks. At the 3-month clinic follow-up visit, the repeat cardiac ultrasound showed a normal origin of LMCA at 2.3 mm (Z score + 1.05) and RCA at 1.6 mm (Z-score +0.14) in diameter.

### Case 2

This is a 2 year and 6-month-old boy, who presents to us for a cough that has lasted more than half a month and a recurrent fever 4 days. Eye conjunctival congestion, dry and chapped lips, strawberry tongue, and rash were noted on admission physical exam. On the third day of admission, a cardiac ultrasound showed enlarge LMCA at 3.58 mm (Z-score +3.66) and RCA at 2.1 mm (Z-score +1.47). The boy was then diagnosed with incomplete KD and was infused with IVIG (2 g/kg/day). 36 h after completing IVIG treatment, the patient exhibited persistent fever, poor spirit, and dyspnea and was then transferred to the PICU. Blood gas showed that the P/F ratio (ratio between the PaO_2_ and the inspired oxygen concentration expressed as a fraction) was <200. The patient underwent endotracheal intubation and mechanical ventilation. Bronchoalveolar lavage fluid was positive for adenovirus and *Mycoplasma pneumoniae*. Fever and hepatosplenomegaly continued to be noted 3 days after transfer to PICU. Laboratory data revealed hemoglobin 79 g/L, platelets 95 × 10^9^/L, serum ferritin 3,594 ng/ml, and triglycerides 3.14 mmol/L. Bone marrow cytology did not detect the hemophagocytic phenomenon. The patient was considered to be IVIG-nonresponsive KD complicated with MAS (Table 1), and a second dose of IVIG was administered after plasma exchange. The patient's clinical symptoms were alleviated. Cardiac ultrasound at the 3-month follow-up showed LMCA at 2.50 mm (Z-score +1.72) and RCA at 1.92 mm (Z-score +1.08).

**Table 1:** Clinical features and laboratory characteristics.

**Case**	**Age (years)**	**Gender**	**Symptoms fit diagnosis of KD**	**Cardiac ultrasound results (maximum coronary diameter)**	**MAS laboratory item**							**hemophagocytic phenomenon**	**Splenomegaly**	**Treatment**	**Time for CAL normal-ization**
					**Fer ng/ml (23.9-336.2)**	**TG mmol/L (<1.7)**	**NK/ul (258–868)**	**PLT 10^**9**^/L (125–350)**	**WBC 10^**9**^/L (4.5–13)**	**HB g/L (120-140)**	**AST U/L (17–59)**				
1	4.8	Female	Fever > 5 days Skin rash Cervical lymphadenopathy Fissure lips Strawberry tongue	LMCA 3.00 mm (Z score + 2.49) RCA 2.69 mm (Z score + 2.39)	10,615	3.42	160	85	14.18	100	37	Yes	Yes	IVIGMPDexVP-16	3 month
2	2.6	Male	Fever > 5 days Conjunctivitis Fissure lips Strawberry tongue Skin rash	LMCA 3.58 mm (Z score + 3.66) RCA 2.10 mm (Z score + 1.47)	3,594	3.14	95	95	2.09	79	99	No	Yes	IVIG	3 month
3	5.1	Male	Fever > 5 days Cervical lymphadenopathy Fissure lips Strawberry tongue	LMCA 2.96 mm (Z score + 2.23) RCA 2.90 mm (Z score + 2.57)	712.6	2.31	213	85	2.82	85	155	No	No	IVIGMP	3 month

*Fer, ferritin; TG, triglyceride; NK, natural killer cell; PLT, platelet; WBC, white blood cell; HB, hemoglobin; AST, aspartate aminotransferase; IVIG, intravenous immunoglobulin; MP, methylprednisolone; VP-16, etoposide; Dex, dexamethasone; CAL, coronary artery lesion*.

### Case 3

This case is a 5 year and 1-month old boy who presented with a history of fever for 5 days and sudden unconsciousness for 2 min (spontaneously recovered afterwards). His highest temperature was 40°C and was associated with chills and occasional cough. Physical examination prominent for red, dry, and chapped lips, strawberry tongue, and enlarged cervical lymph node (1.5 cm). An electroencephalography (EEG) was abnormal (large number of spike-waves/slow spike-waves activated in the right central region, frontal area, right center, and central midline during admission). Given continued fever, atypical KD was suspected, and a cardiac ultrasound was performed on the fourth day of admission and showed LMCA at 2.96 mm (Z-score +2.23) and RCA at 2.90 mm (Z-score +2.57). The patient was diagnosed with incomplete KD and was administered IVIG (2 g/kg/d). As fever persisted 36 h after the IVIG treatment, further work up with lab studies showed white blood cell count 2.94 × 10^9^/L, hemoglobin 88 g/L, platelets 78 × 10^9^/L, triglycerides 2.31 mmol/L, and serum ferritin 712.60 ng/ml. Bone marrow cytology did not detect the hemophagocytic phenomenon. The patient was diagnosed with incomplete KD complicated with MAS (Table 1, according to sJIA-MAS 2016). The patient then received a second dose of IVIG (2 g/kg/d) and 3 days of IV methylprednisolone (30 mg/kg/d), followed with improvement of symptoms and resolution of fever. His cardiac ultrasound at the 3-month follow-up visit showed LMCA at 2.26 mm (Z-score +0.77) and RCA at 2.02 mm (Z-score +1.01).

## Discussion

KD is a common vasculitis in children, and its incidence is highest among children living in East Asia or Asian children living in other regions of the world (14). Clinical manifestations of KD primarily include systemic inflammation and skin mucosal inflammation. The diagnosis and treatment guidelines by the American Heart Association in 2017 proposes a diagnosis of KD based on more than 5 days of fever and ≥4 main clinical characteristics. If a child has fever for ≥5 days and meets 2 or 3 main clinical characteristics combined with coronary artery dilatation, the child can also be diagnosed with KD (incomplete KD), except for exudative conjunctivitis, exudative pharyngitis, ulcerative stomatitis, bullous or vesicular rash, and systemic lymphadenopathy or splenomegaly (3, 15).

The three cases in our report all met the clinical diagnostic criteria for incomplete KD. Cardiac ultrasound of all three showed coronary artery dilatation, among which two cases met the criteria for a small coronary artery aneurysm (3). All three patients have persistent fever at 36–48 h after administration of a single dose of IVIG at 2 g/kg, indicating non-responsiveness to IVIG treatment. In this study, 10 days after onset, the disease progressed rapidly and manifested as persistent fever, hepatosplenomegaly, pancytopenia, significant elevation of ferritin and hypertriglyceridemia. These symptoms are well consistent with clinical features and laboratory characteristics of MAS. Thus, the three cases presented here met the diagnosis of IVIG-nonresponsive KD complicated with MAS.

The incidence rate of KD complicated with MAS is 1.1–1.9% (9, 10). The risk of KD complicated with MAS increases 7-fold in KD patients over the age of 5 years old (16). Eighty-five percent of KD occurs in children under the age of 2 years old. In this report, all three patients were over 2 years old and older than the common age of KD onset. Kato et al. found that 49.3% of patients with KD complicated with MAS were over 5 years old (17).

MAS may occur in any stage of KD (acute stage, subacute stage, or recovery stage) and may also occur prior to a KD diagnosis, but in most cases, it appears simultaneously with KD (12). The MAS in the three cases of this study also presented simultaneously with KD. The mortality rate of MAS is 8–22% (18, 19), and prompt treatment is the key positive factor for survival of patients with MAS. Therefore, earlier diagnosis of MAS in children with KD is of most importance for prompt treatments and better prognosis. The early diagnosis of KD complicated with MAS is a clinical challenge as MAS lacks specific diagnostic features and has many overlapping manifestations and laboratory findings with KD. For example, persistent fever, skin rash, thrombocytopenia, abnormal liver enzymes, anemia (20), and hypertriglyceridemia can be found both in KD and MAS. However, previous reports showed KD patients with ICU admission tended to have significant anemia and thrombocytopenia (21, 22). Thrombocytopenia was common in the acute critical stage, whereas thrombocytosis was thought to be a feature of later phase. Early thrombocytopenia was considered a risk marker for coronary aneurysm formation (2). Despite the overlap symptoms between KD and MAS, a serial dynamic echocardiogram test was crucial for differentiation and diagnosis especially when the two diseases occurred together (23). Our study suggests that clinical high vigilance and frequent surveillance of MAS is very helpful for diagnosis of this entity, especially in setting of fever persisting more than 10 days or IVIG-nonresponsive KD patients.

The diagnosis criteria for rheumatic diseases complicated with MAS are mainly provided by the HLH-2004 diagnostic criteria established by the International HLH Association (24). Furthermore, the HLH-2004 criteria cannot be applied for the clinical application of secondary HLH, including sJIA complicated with MAS (25). The European League Against Rheumatism (EULAR) together with the American College of Rheumatology (ACR) published the diagnostic criteria for sJIA complicated with MAS as sJIA-MAS (26), which also suggested an application for KD complicated with MAS (27). For febrile patients with confirmed or suspected sJIA and ferritin > 684 ng/ml, the sJIA-MAS 2016 suggests that they can be diagnosed with sJIA-MAS as long as they meet any 2 or more of the following criteria: platelet count ≤ 181 × 10^9^/L, aspartate aminotransferase > 48 U/L, triglycerides > 1,560 mg/L, and fibrinogen ≤ 3,600 mg/L. In this study, cases 1 and 2 met the diagnostic criteria for HLH-2004, and case 3 met the diagnostic criteria for sJIA-MAS 2016. However, the sJIA-MAS 2016 diagnostic criteria were published recently, and more clinical applications are still needed before a final determination.

Approximately one-third of HLH patients have nervous system abnormalities, including seizures, altered mental status, and ataxia (28). Those neurological manifestations can either be the main reason for seeking medical care or sometimes as an initial clinical presentation (29). Magnetic resonance imaging (MRI) of the brains of HLH patients may show low-density or necrotic areas (30). Cerebrospinal fluid abnormality could be seen in 50% of the patients, and their risk of neurological sequelae increases (31). Altered consciousness and abnormal EEG occurring prior to KD symptoms such as in case 3 of our study, is similar to previous reports (29).

Although hemophagocytic phenomenon can be used as a marker for the overactivation of macrophages, it is not a specific manifestation of HLH and does not always occur in the early stage of MAS. Furthermore, bone marrow aspiration is an invasive procedure that is not included in the sJIA-MAS 2016 (26). Hemophagocytic phenomenon could not be found in any of the MAS cases (25–100%) from bone marrow examination (32). Some patients may experience the hemophagocytic phenomenon in the late stage of the disease after clinical symptoms are alleviated (23). In our patients, bone marrow cytology examinations showed a negative finding of hemophagocytic phenomenon in all three cases, but they fit the diagnosis of MAS. MAS can be diagnosed if patients meet clinical and laboratory characteristics, even in the absence of the hemophagocytic phenomenon in the bone marrow.

Splenomegaly is rarely present in patients with KD but occurs in 69% of patients with KD complicated with MAS (12). KD patients should be considered for the possibility of MAS if persistent fever and presence of splenomegaly are noted (12). Thrombocytopenia is a high risk factor for acute myocardial infarction or coronary aneurysm in KD patients (33). In our patients, all three had thrombocytopenia and coronary artery dilatation, of which two had small coronary aneurysms. The coronary arteries of all three patients regressed to normal range within 3 months after disease onset. The findings of this report indicate a high incidence rate (100%) of coronary abnormality in patients with KD complicated with MAS and a high regression rate following appropriate treatment were observed.

A summary of this case report of three KD patients complicated with MAS shows its following clinical features: (1) more common in male gender patients, that is consistent with the report of literature (34); (2) more likely in patients older than 2 years old than patient with typical KD who are less than 2 years old; (3) fever persistent more than 10 days; (4) splenomegaly; (5) non-responsiveness to IVIG; and (6) incomplete KD.

MAS is a rare complication of KD with serious clinical implications. It commonly occurs with coronary involvement. Additional treatment should be given to patients who are not completely responsive to the initial IVIG treatment, including a second course of IVIG, glucocorticoid, tumor necrosis factor (TNF) inhibitor, immunosuppressant agents, or plasma exchange. Both HLH-2004 or sJIA-MAS 2016 has provided diagnosis criteria for KD with MAS, even when bone marrow aspiration has negative findings.

## Data Availability Statement

All datasets generated for this study are included in the manuscript/supplementary files.

## Ethics Statement

The studies involving human participants were reviewed and approved by the Ethics Committee of Baoan Maternal and Child Health Hospital. Written informed consent to participate in this study was provided by the participants' legal guardian/next of kin. Written informed consent was obtained from the individual(s), and minor(s)' legal guardian/next of kin, for the publication of any potentially identifiable images or data included in this article.

## Author Contributions

PJ observed the patients, collected and analyzed the data, and drafted the manuscript. YL and JX helped to observe the patients. XL participated in writing the study. CL coordinated the execution of the studies and corrected the manuscript. All authors read and approved the final manuscript.

### Conflict of Interest

The authors declare that the research was conducted in the absence of any commercial or financial relationships that could be construed as a potential conflict of interest.
